# Chlorhexidine-alcohol versus povidone iodinealcohol antisepsis for catheter-related infection prevention: an open-label, multicentre, randomised controlled trial

**DOI:** 10.1186/2197-425X-3-S1-A409

**Published:** 2015-10-01

**Authors:** O Mimoz, J-C Lucet, T Kerforne, J Pascal, B Souweine, V Goudet, A Mercat, L Bouadma, S Lasocki, S Alfandari, A Friggeri, F Wallet, N Allou, S Ruckly, D Balayn, A Lepape, J-F Timsit, CLEAN Investigators

**Affiliations:** Surgical ICU, Poitiers, France; Bichat University Hospital, Infection Control, Paris, France; Poitiers, Surg ICU, Poitiers, France; University Hospital Gabriel Montpied, Surg ICU, Clermont-Ferrand, France; Hopital NeckerGabriel Montpeid, Clermont-Ferrand, France; Maladies Infectieuses, Université Poitiers, Poitiers, France; CHU Angers, Med ICU, Angers, France; Medical and Infectious Diseases ICU, Université Paris Diderot, Paris, France; CHU Angers, Surg ICU, Angers, France; CH Tourcoing, ICU, Tourcoing, France; Université Lyon Sud, Surg ICU, Lyon, France; Université Lyon Sud, Lyon, France; Université Paris Diderot, Surg ICU, Paris, France; OUTCOMEREA-INSERM UMR 1137, Descision Science in Infectious Diseases (DesCID), Paris, France; Surg ICU, Université Poitiers, Poitiers, France; Paris, Medical and Infectious Diseases ICU, Paris, France

## Introduction

Catheter-related infections are common life-threatening healthcare-associated infections whose incidence can be decreased by improving the quality of care. Optimisation of skin antisepsis is essential to prevent short-term catheter-related infections. We hypothesised that chlorhexidine-alcohol was more effective than povidone iodine-alcohol as a skin antiseptic for preventing infections.

## Methods

In our open-label, randomised controlled trial, we enrolled consecutive adults (≥ 18 years) admitted to one of 11 French intensive care units and requiring at least one central-venous, haemodialysis, or arterial catheter. Before catheter insertion, we randomly assigned patients by a secure web-based random-number generator (1:1:1:1, permuted blocks of eight, stratified by centres) to have all the intravascular catheters prepared with chlorhexidine-alcohol or povidone iodine-alcohol, applied in one or two steps (cleaning the skin with antiseptic detergent prior to antiseptic application). The incidence of catheter-related infections with chlorhexidine-alcohol versus povidone iodine-alcohol was the primary outcome. Physicians and nurses were not masked to group assignment but microbiologists and outcome assessors were. Analyses were by intention to treat. This study is registered with ClinicalTrials.gov, number NCT01629550 and the protocol has been published (see Goudet et al).

## Results

Between Oct 26, 2012, and Feb 12, 2014, 2546 patients were eligible to participate in the study and 2349 (92.3%) were randomly assigned to chlorhexidine-alcohol (1181 patients [2547 catheters]) or povidone iodine-alcohol (1168 patients [2612 catheters]). Chlorhexidine-alcohol was associated with lower incidence of catheter-related infections (0.28 versus 1.77 per 1000 catheter-days; hazard ratio 0.15, 95% CI 0.05,0.41; p = 0·0002) compared to povidone iodine-alcohol. None of the antiseptics caused systemic adverse events. Chlorhexidine-alcohol was also superior for secondary end points:(CR-BSI HR = 0.21 (0.07,0.59), p = 0.003 and catheter colonization HR = 0·18 (0.13,0.24), p < 0.0001).

Detersion prior to antisepsis did not influenced the risk of infection (HR= 1.03 (0.57,1.88), p = 0.91) or catheter colonization (HR= 1.10 (0.89,1.35), p = 0.39).

Severe skin reactions occurred more frequently with chlorhexidine-alcohol (27 [2.6%] versus 7 [0.7%] patients; p = 0.0017), and led to chlorhexidine discontinuation in two patients.

## Conclusions

For skin antisepsis, chlorhexidine-alcohol provides greater protection against short-term catheter-related infections than does povidone iodine-alcohol, and it should be included in all bundles for prevention of intravascular catheter-related infections.

## Grant Acknowledgment

Unrestricted research grant from Carefusion
